# Understanding implementation breakdown: lessons from a type 2 hybrid effectiveness-implementation study in residential aged care

**DOI:** 10.3389/frhs.2026.1813706

**Published:** 2026-10-26

**Authors:** Sharon Andrews, Kelly Edwards, Christine Stirling, David Lees, Pieter Van Dam, Maryanna Klatt, Jacqueline Caputo, Rachel Kornhaber, Peter Williams

**Affiliations:** 1 School of Nursing, University of Tasmania, Sydney, NSW, Australia; 2 School of Nursing, University of Tasmania, Hobart, TAS, Australia; 3 Family and Community Medicine, The Ohio State University, Columbus, OH, United States; 4 School of Health—Nursing, University of New England, Armidale, NSW, Australia; 5 OneCare Ltd, Hobart, TAS, Australia

**Keywords:** implementation science, implementation barriers, implementation strategies, Consolidated Framework for Implementation Research (CFIR), Nursing homes, co-design, mindfullness-based interventions

## Abstract

**Introduction:**

Australia's aged care sector faces mounting pressures driven by an ageing population, increasing care complexity, and persistent workforce shortages. Burnout among residential aged care staff is a critical issue influencing retention, care quality, and staff wellbeing. Despite evidence supporting mindfulness-based interventions in healthcare, their uptake in Residential Aged Care Services (RACS) remains limited. This Type 2 hybrid effectiveness-implementation study examined the feasibility, acceptability, appropriateness, and effectiveness of Mindfulness in Motion (MIM) program within an Australian aged care organisation.

**Methods:**

The study involved three stages: (1) capacity building through a Mindfulness Action Group (MAG); (2) co-design of an implementation strategy informed by the Consolidated Framework for Implementation Research and aligned with Expert Recommendations for Implementing Change; and (3) program implementation with a mixed-methods evaluation across five residential aged care sites. Quantitative measures of burnout, stress, resilience, and work engagement were collected at baseline (T1) and completion (T2). Qualitative data addressing implementation outcomes were derived from interviews with MIM participants and post-program meetings with MAG members. Trial registration: ACTRN12623000795695.

**Results:**

Adoption of the MIM program was low: 22 staff registered and 11 commenced the Stage 3 program. Two of these participants, together with six MAG members who had completed the same pre- and post-program measures, provided complete paired T1-T2 data (*n* = 8). Exploratory analyses indicated some favourable trends in staff wellbeing; however, the small, self-selected sample precluded definitive conclusions regarding program effectiveness. Interview data indicated that MIM was acceptable and perceived as beneficial by participants. However, intersecting inner- and outer-setting barriers, including chronic staffing shortages, heavy workloads, scepticism about implementation support, limited middle-management engagement, and communication silos, undermined adoption, feasibility and appropriateness.

**Discussion:**

The MIM program was viewed as acceptable by aged care staff participants but could not be implemented as intended due largely to entrenched workforce and organisational barriers. Theoretically informed, co-designed strategies proved insufficient when contextual barriers were resistant to change or emerged only during implementation. Iterative, adaptive approaches that address structural pressures, staff perceptions, management engagement, and communication needs are required to support sustainable wellbeing interventions in aged care.

## 1 Introduction

Residential aged care services (RACS) provide 24-hour accommodation, nursing, personal care and support to older adults who can no longer live independently. Aged care staff are exposed to ongoing workplace stressors, including staffing shortages, high workloads, and the complex needs of residents and their families. As a result, burnout has emerged as a significant concern within the residential aged care workforce (1, 2). In 2020, in Australia, 277,671 people were employed in residential aged care, including 208,903 direct care workers of which 70% were Personal Care Workers, 23% nurses, and 7% allied health professionals (3). The pressures on aged-care staff need to be addressed as Australia's ageing population continues to drive demand for aged care services. In 2022, people aged 65 years and over comprised 16% of the Australian population, a proportion projected to reach up to 23% by 2066 (4). By 2050, an estimated 3.5 million Australians will require aged care support, with a workforce demand approaching one million aged care workers (4).

The sector already faces critical challenges in recruitment, retention, and job satisfaction (5). Each year an estimated 65,000 employees leave the workforce, and over one-third of the current workforce plan to leave within five years (6). Staff stress, absenteeism, and burnout are known factors influencing retention, care quality, and staff wellbeing (6, 7). At the same time, the increasing availability of home-based support services means older adults often enter RAC later with greater frailty and more complex care needs (1). This increases the skills and workforce capacity required to manage higher levels of resident dependency and intensifies existing work demands, contributing to burnout (8). Given that RAC represents one of the largest and fastest-growing sectors, addressing staff burnout is critical (8). It is well understood that organisational culture, policy reforms (9) and personal attributes such as self-esteem and commitment shape experiences of stress and burnout (2). Burnout is a psychological response to prolonged occupational stress (10, 11) characterised by emotional exhaustion, depersonalisation, and a diminished sense of accomplishment (11, 12). It is associated with decreased empathy, cynicism, and reduced work performance (13, 14), ultimately affecting care quality, productivity, and staff turnover (7).

Evidence suggests that creating supportive work environments (1) and training in self-care and coping strategies can help mitigate burnout (15, 16). Mindfulness interventions cultivate present-moment awareness through meditation, gentle movement, and emotional regulation. Across a range of healthcare settings these interventions have demonstrated enhanced self-compassion, improved wellbeing, and decreased burnout among health professionals, including nurses and physicians (17–21).

The Mindfulness in Motion (MIM) program integrates mindfulness practice with gentle yoga, reflective exercises and relaxing music. The movement component facilitates embodied mindfulness and is central to the program (22). Studies in high-stress healthcare environments, including surgical and neonatal intensive care units, have reported reductions in perceived stress and burnout and improvements in sleep, mood and resilience (22–25). At one US medical centre, MIM was associated with a 27% reduction in burnout prevalence and improved resilience and work engagement (26). An online format developed during the COVID-19 pandemic demonstrated outcomes comparable with face-to-face delivery (27). Its workplace focus, combination of mindfulness and gentle movement, evidence among high-stress healthcare workers and capacity for online delivery across multiple sites made MIM a plausible intervention for the residential aged care workforce.

Evidence for mindfulness interventions in RAC remains limited, although one trial among nurses caring for older people with dementia in residential care reported reductions in compassion fatigue and burnout (28). To our knowledge, no previous study has evaluated MIM or examined its implementation in residential aged care (RAC). This represents an important evidence gap because the distinctive workforce and organisational conditions of this sector may influence both program outcomes and implementation feasibility. Research is therefore needed to determine whether MIM is effective, acceptable, appropriate and feasible within routine RAC practice.

This focus on implementation is important because the same workforce and organisational conditions that create the need for staff wellbeing interventions, particularly chronic staffing shortages, high workloads and workplace stress, may also impede their implementation (29–31). In RAC, competing care responsibilities and limited workforce capacity can reduce staff availability to participate in new initiatives and constrain organisational readiness for change (30). Workload demands, competing clinical responsibilities, and insufficient workforce capacity have been identified as major barriers to implementation, whereas supportive leadership, education, peer champions, and effective communication may facilitate adoption (32). Therefore, understanding contextual factors that support or impede the implementation is essential when assessing the effectiveness of programs targeted at improved staff wellbeing.

Implementation science has become increasingly important in regard to the Australian aged care sector. The Royal Commission into Aged Care Quality and Safety highlighted the need to increase the translation of research evidence and innovative practices in aged care, as part of a broader reform agenda (5). However, evidence to guide implementation in this sector remains limited. RAC differs from other care sectors in its workforce composition, resource constraints, culture and organisational structures (29, 30). Therefore, implementation strategies developed in other healthcare settings may not transfer directly to this context. Accordingly, implementation frameworks, such as the Consolidated Framework for Implementation Research (CFIR), have been shown to support the systematic identification of multilevel contextual barriers and facilitators that can inform implementation planning in RAC (31, 33).

This study aimed to evaluate the implementation and effectiveness of the eight-week online MIM program (22, 34) across five residential aged care services operated by one Australian aged care organisation. A Type 2 hybrid effectiveness–implementation approach was selected because MIM had demonstrated promising effectiveness in other healthcare settings but had not previously been evaluated in residential aged care. This approach enables the concurrent examination of intervention effectiveness and implementation outcomes. By examining these dimensions simultaneously, Type 2 hybrid studies can identify contextual barriers and facilitators that may influence both implementation and observed intervention outcomes, thereby supporting more timely translation into routine practice (35). Consistent with this approach, the study examined staff wellbeing outcomes alongside implementation outcomes, such as adoption, acceptability, appropriateness and feasibility.

## 2 Methods and materials

This study used a Type 2 hybrid effectiveness-implementation design and was guided by a registered project protocol (ACTRN12623000795695) listed on the Australian New Zealand Clinical Trials Registry. The intervention component comprised the eight-week online MIM program, while the implementation component comprised a co-designed implementation strategy. Effectiveness outcomes included burnout, perceived stress, work engagement, resilience, sick leave and staff turnover. Implementation outcomes included adoption, acceptability, appropriateness and feasibility. The hypotheses were:

Hypothesis 1: That participation in the MIM program would reduce perceived stress and burnout and increase resilience and work engagement.
Hypothesis 2: That participation in the MIM program would reduce staff sick leave and turnover.

The project was structured into three sequential stages encompassing staff capacity-building, co-design of implementation strategies, and program delivery with mixed-methods evaluation. Ethical approval was obtained from the University of Tasmania Human Research Ethics Committee (Project ID 29059).

### 2.1 Stage 1: capacity building and preparation (months 1–6)

Six staff members representing diverse professional roles across the aged care organisation's 5 sites were recruited as project champions, collectively known as the *Mindfulness Action Group* (MAG) (See Table 1). All members completed the MIM program delivered through Ohio State University (OSU) by trained facilitators affiliated with the program. Three members of the MAG subsequently undertook facilitator and fidelity training provided by OSU. These three staff were responsible for delivering the MIM program in the aged care service.

**Table 1:** MAG members.

MAG member ID	Role
MAG 1	Clinical Nurse Manager
MAG 2	Operations Manager
MAG 3	Work Health and Safety Advisor
MAG 4	Personal Care Assistant Team Leader
MAG 5	Work Health and Safety Advisor
MAG 6	Personal Care Assistant

### 2.2 Stage 2: co-design of the implementation strategy (months 3–7)

The MAG collaborated with the research team across four co-design workshops facilitated by SA and KE. MAG members contributed detailed knowledge of local workflows, staffing, communication and leadership, while the research team structured discussion using the Consolidated Framework for Implementation Research (CFIR) (33). These co-design meetings were audio-recorded. After each workshop, the facilitators prepared a written summary of identified barriers, enablers and proposed implementation components; MAG members reviewed the summary and refined it at the next workshop. Decisions were made through facilitated discussion and iterative agreement, and the implementation plan was progressively refined until the group considered the strategies locally feasible. Strategies spanned three CFIR domains (33): Intervention Characteristics, Inner setting and Process. The final strategies were mapped to the Expert Recommendations for Implementing Change (ERIC) matching tool (36) to confirm alignment with evidence-based approaches. The resulting CFIR determinants and implementation strategies are presented in Table 2.

**Table 2:** CFIR mapped implementation barriers, enablers and corresponding strategies.

CFIR domain	CFIR construct	Implementation barrier (B)/facilitator (F)	Co-designed strategy
Intervention characteristics	Adaptability	(B) Reflective activities in the program considered by staff to be confronting, lack of clarity that reflection was optional may impact engagement.	Develop clear steps for MIM facilitators to ensure MIM participants know sharing/reflection is optional.
Inner setting	Networks & Communication	(B) Limited informal cross-site communication that leads to information silos	Emails sent to all staff to raise awareness. Targeted face-to-face awareness raising sessions at each of the services to promote project participation.
	Culture	(F) Wellness focus on staff wellness	Leverage facilitator by including information about the MIM program in the Wellness newsletter and on the Wellness notice boards.
		(B) Hierarchical dynamics may inhibit open reflection when managers are present; trust-building needed.	MIM facilitators to reinforce that reflection sessions are optional and everyone has a voice in the sessions.
	Implementation Climate—Compatibility	(B) High workloads and staffing pressures may undermine perceived fit with daily work.	Provide backfill for staff who are attending the MIM session. Run MIM sessions at 07:00 and 14:00 hrs so that they are around shift-changeover times and can facilitate attendance with workflow.
	Implementation Climate—Relative Priority	(B) Competing operational demands risk de-prioritisation; staff need reassurance about shift coverage to attend.	Emails, posters and project information sheet all reinforce that attendance at the program is supported by management and that staff time will be covered/paid attendance.
	Readiness for Implementation—Leadership Engagement	(F) Strong support from the CEO of the organization for the project.	Emails sent from CEO to all staff to encourage and legitimize participation.
	Readiness for Implementation—Leadership Engagement	(B) Insufficient middle-management support may limit staff ability to participate in the program and/or may discourage staff participation.	CEO to provide briefings to middle management to support program. MAG members with management roles actively promote the program to other middle managers and encourage them to support their staff.
	Readiness for Implementation—Available Resources	(B) Staffing shortages will be a barrier to staff willingness to engage with the MIM program.	Provide clear communication that staff will be paid for their time and backfilled to attend via staff information face-to-face awareness raising sessions at each service, project posters in staff areas and letters of invitation.
Process	Opinion Leaders	(F) Recruitment and training of the MAG group to support the project and supportive CEO who had regular contact with project team.	MAG members and CEO act as internal opinion leaders communicating expectations, encouraging participation, and reinforcing organisational support.

### 2.3 Stage 3: program delivery and evaluation (months 8–10)

Three trained MAG facilitators delivered the 8-week MIM program across five residential aged care sites within one organisation. Each cohort attended one synchronous, one-hour online session per week. Sessions included reflective writing, a brief didactic component, optional sharing, guided mindfulness meditation and gentle yoga, and a closing meditation. Participants were also encouraged to complete 10–20 min of individual practice on at least five days each week. Participants could choose from a 7 am session or a 2 pm session. These session times aligned with shift changeovers. Dedicated rooms were available at each site so staff could join before, after or during their shifts. At every session, a second trained facilitator completed a session-level fidelity check and privately prompted the lead facilitator if a core component had been missed.

A mixed-methods evaluation captured effectiveness outcomes (burnout, perceived stress, resilience, work engagement, sick leave and turnover) using a single pre-post test design and implementation outcomes (adoption, acceptability, appropriateness, and feasibility) using qualitative experiential data (Table 3).

**Table 3:** Effectiveness and implementation data collection.

Dimension	Outcome	Data collection tools	Data collection time points^a^
Effectiveness (primary outcomes)	Burnout	Maslach Burnout Inventory, comprising three subscales Emotional exhaustion, depersonalization and personal accomplishment. (MBI) (12)	T1 and T2; planned T3 and T4 collection abandoned
	Staff perceived stress	Survey tool: perceived Stress Scale (PSS) (38)	
	Resilience	Connor-Davidson Resilience Scale (CD-RISC) (40)	
	Work engagement	the Utrecht Work Engagement Scale (UWES), including the Vigor, Dedication, and Absorption subscales (39)	
	Staff sick leave and turnover	Organizational audit data of sick leave and staff turnover	T3 and T4 (collection abandoned)
Adoption	Uptake and engagement with the MIM program	% Attendance at MIM sessions over eight-week period	T1 and T2
Appropriateness, Feasibility, acceptability.		MIM participant interviews MAG post-program meeting data.	T3

a Data collection time points pre-intervention baseline (T1), post-program completion (T2), 2-month follow-up (T3), and 12-month follow-up (T4).

### 2.4 Setting and participants

The study was conducted in partnership with one Australian aged care organisation operating five residential aged care services (RACS) with a combined workforce of approximately 1,000 staff. The organisation is a not-for-profit provider offering 24-hour nursing and personal care across both regional and metropolitan sites.

Eight potential MAG members were identified by the organisation's executive because they held leadership or practice roles and had at least two years' organisational experience. The project team sent each candidate an invitation and participant information and consent form through the organisation's administrative channels. Participation was voluntary; six staff self-nominated and provided written consent. Their professional roles are presented in Table 1.

MIM participants were eligible if they were employed part-time or full-time at a participating site, provided direct care or resident support, or held a decision-making role influencing care delivery, and were willing and able to attend the 8-week program and able to complete program measures. Casual staff and staff unable to meet these participation requirements were excluded. The protocol set a pragmatic target of 75 MIM participants (five cohorts of up to 15, the maximum recommended program group size). This target was based on program delivery capacity and guidance from the MIM program developers regarding maximum group size rather than a formal power calculation.

Recruitment invitations and participant information and consent forms were distributed to staff by a third party at the organisation through staff email so that the research team did not receive staff contact details. Recruitment was reinforced through CEO communications, site information sessions, the staff wellness newsletter, posters and a QR-code expression-of-interest link. Interested staff contacted the research team directly, eligibility was confirmed, and written informed consent was obtained. Staff were advised that participation was voluntary and that non-participation would not affect their employment. No separate research incentive was offered; paid release from duties and backfill were planned to support attendance during rostered hours. Service managers were notified only after consent so that rostering could be arranged.

### 2.5 Data collection

Stage 3 data sources included quantitative questionnaires, qualitative interviews with MIM participants (staff who were not MAG members), and post-program MAG meetings. Data collection was structured around Proctor et al.'s (37) implementation outcomes taxonomy to capture intervention effectiveness and key implementation outcomes (Table 3). Program effectiveness was assessed using paired pre-post data from all staff who completed the MIM program, including MAG members who undertook MIM through Ohio State University. Pooling of MAG and MIM participants was restricted to the exploratory effectiveness analysis because both groups received the same 8-week core curriculum and completed the same measures immediately before and after their first MIM exposure. Program fidelity for the MAG and MIM participants was monitored through a standardised curriculum and session-level fidelity checks. However, MAG and MIM participants were not combined for data collection on adoption or other implementation outcomes because MAG participation was role-based and project-driven.

Program adoption was assessed only among Stage 3 MIM participants because the program was being newly introduced into the setting. Consistent with Proctor et al.'s definition, adoption was conceptualised as uptake and actual use of the program within the implementation site. Program effectiveness measures were planned at four time points (T1-T4); however, due to low response rates, only baseline (T1) and immediate post-intervention (T2) quantitative data were collected. Participants who completed MIM were invited to interviews at T3 to explore perceived benefits of MIM, acceptability of the program, factors facilitating or hindering recruitment and attendance, fit with work routines, and suggestions for improvement. MAG members also met twice to examine the acceptability of MIM, and the appropriateness, and feasibility of the implementation strategies. Meeting prompts examined the barriers and enablers to recruitment and on the ground delivery of the program, and modifications required for future implementation.

#### 2.5.1 Departures from the registered protocol

The protocol planned approximately 75 Stage 3 participants, quantitative data collection at T1-T4, and organisational sick-leave and turnover data. Recruitment and paired-data completion were substantially lower than anticipated. Because T2 collection required extensive follow-up and further collection would have imposed additional burden on participants and the partner organisation for limited analytical value, T3 and T4 quantitative data collection and organisational sick-leave and turnover data collection were discontinued.

### 2.6 Data analysis

#### 2.6.1 Quantitative data

Quantitative data were analysed using a pre-post repeated-measures approach. Descriptive statistics (means and standard deviations) were calculated at baseline and follow-up for burnout [Maslach Burnout Inventory [MBI] (12)], perceived stress [Perceived Stress Scale [PSS] (38)], work engagement [Utrecht Work Engagement Scale [UWES] (39)], and resilience [Connor-Davidson Resilience Scale [CD-RISC] (40)], according to the scoring guidance for each instrument. Changes between T1 and T2 were analysed using one-tailed paired-samples t-tests. One-tailed tests were selected because the study hypotheses specified the anticipated direction of change before analysis, with statistical significance set at *p* < 0.05. Given the small sample size, effect sizes were calculated using repeated-measures standardised mean differences (Hedges' *g*_rm_), assuming a conservative within-person correlation of r = 0.50. Ninety-five per cent confidence intervals were two sided and were calculated for all effect sizes to support interpretation of precision and uncertainty. Analyses included participants with complete paired observations only and were conducted in the Statistical Package for the Social Sciences (SPSS), version 31.

#### 2.6.2 Qualitative data

Post-program MAG meetings (*n* = 2) and interviews with MIM participants (*n* = 4) were conducted by SA and analysed using a qualitative framework analysis approach (41). A preliminary coding framework combined deductive categories drawn from CFIR determinants (42) and Proctor implementation outcomes with inductive codes for unanticipated barriers, enablers and perceived program benefits. SA read the transcripts, developed and applied the initial coding framework in NVivo, and compared patterns across MIM participant and MAG data. KE reviewed the coding framework, coded extracts and developing categories. Differences were resolved through discussion and re-examination of the source data, with clarification sought from MAG members when required. Codes were grouped into higher-order analytic categories that structured the findings. Trustworthiness was supported through the criteria of credibility, dependability and confirmability (43, 44). Credibility was strengthened through comparison of patterns across MIM participant and MAG data sources and the use of verbatim quotations to demonstrate that interpretations were grounded in participants' accounts. Dependability was supported through a documented audit trail of coding and analytic decisions maintained in NVivo. Confirmability was supported through second-investigator review of the coding framework and coded extracts, resolution of differences through discussion, and re-examination of the source data to ensure that the findings could be traced back to participants' accounts.

Interview prompts explored perceived benefits and drawbacks, acceptability, fit with work routines, factors affecting attendance and suggestions for improvement; MAG prompts additionally examined adherence to the implementation plan and barriers, enablers and adaptations during delivery.

## 3 Results

### 3.1 Stage three: implementation/program delivery and evaluation

#### 3.1.1 Results for measures of adoption and effectiveness

Across both phases of the project, 28 staff were enrolled to receive MIM: six MAG members in the capacity-building phase and 22 staff who registered during Stage 3 of the project rollout. However, adoption was assessed for the Stage 3 rollout only, because MAG participation was role-based and occurred during the earlier capacity-building phase rather than representing routine program uptake. Approximately 1,090 staff received a Stage 3 invitation; 22 registered (2.0% of those invited), and 11 commenced the MIM program across the two cohorts offered. Poor uptake occurred despite the strategies in the implementation plan, including face-to-face information sessions, invitations from the CEO, promotion in the staff wellness newsletter with a QR-code link, and posters across sites. Attendance among the 11 participants was variable: one attended one session (12%), four attended four sessions (50%), two attended five sessions (63%), three attended seven sessions (88%), and one completed all eight sessions (100%).

For the exploratory effectiveness analysis, data from MAG and Stage 3 participants were combined because both groups completed the same standardised eight-week MIM program and the same outcome measures at comparable pre- and post-program time points. Of the 11 Stage 3 participants, two completed both baseline (T1) and post-program (T2) surveys. All six MAG members had complete paired data, yielding an exploratory effectiveness sample of eight: six MAG members and two Stage 3 participants (See Figure 1, Participant Flowchart). The complete-case sample included staff in clinical roles (two enrolled nurses), direct-care roles (two personal care assistants), operational roles (one clinical manager and one operations manager), and corporate service roles (two human resources/quality and safety staff). Further disaggregation by role, site or demographic characteristics was not undertaken because the very small sample sizes would be uninformative and could compromise confidentiality.

**Figure 1:**
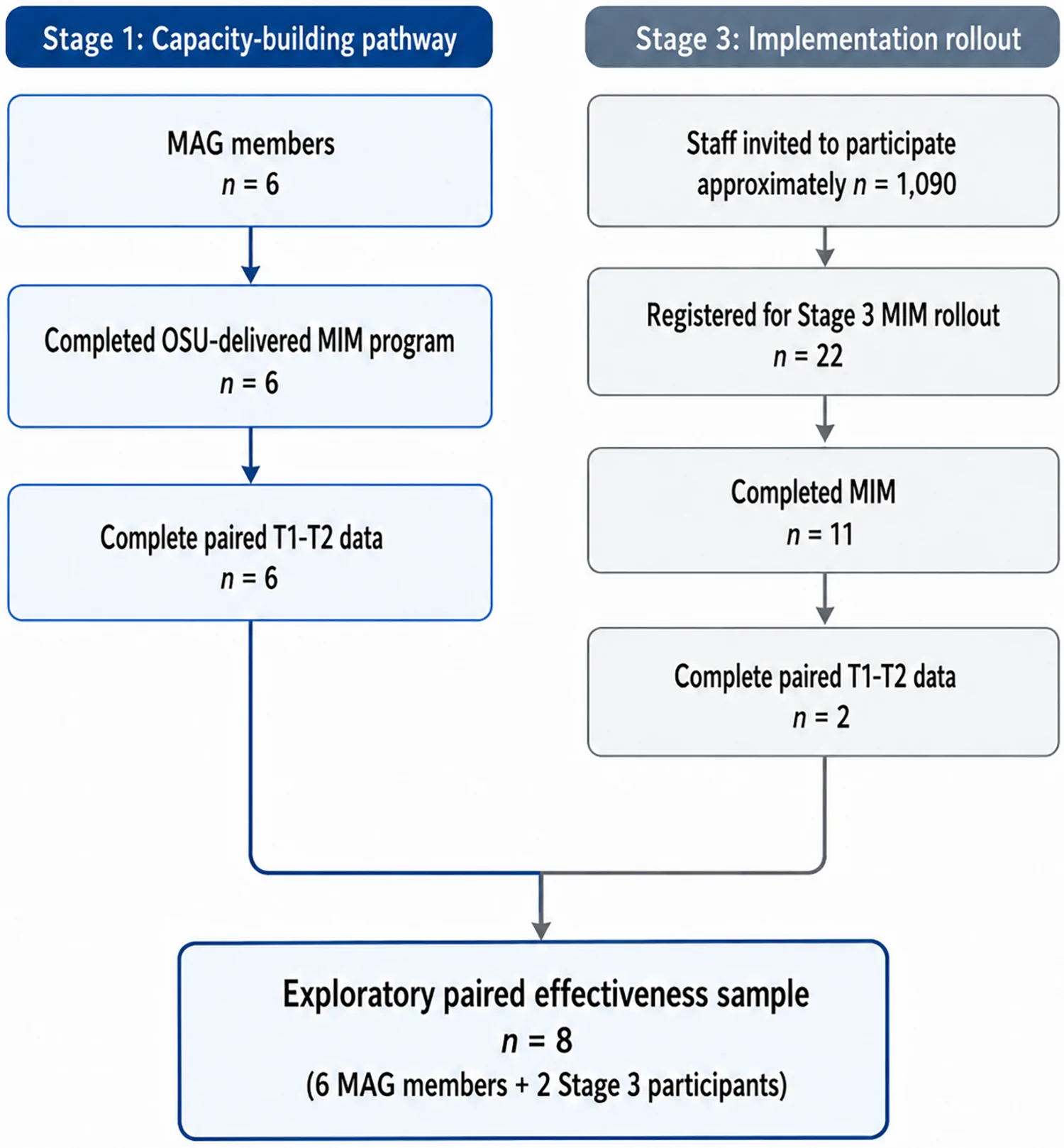
Participant flow through mindfulness in motion program.

Quantitative analyses suggested mixed patterns across staff wellbeing outcomes following completion of the MIM program (Table 4). For burnout, mean scores showed small reductions in emotional exhaustion and depersonalisation, which were in the hypothesised direction, whereas personal accomplishment decreased, which is not consistent with reduced burnout. Effect size estimates for the burnout subscales were small to moderate and not significant, with confidence intervals crossing zero. In contrast, work engagement increased significantly from baseline to follow-up (*p* = 0.02), with a moderate-to-large effect size (Hedges' *g*_rm_ = −0.79). At the subscale level, dedication scores increased significantly (*p* = .015; *g*_rm_ = −0.858) representing a large effect. Vigor (*p* = .03; *g*_rm_ = −0.69) showed statistically significant improvements, however the confidence interval crossed zero, suggesting considerable uncertainty of the effect around the effect size estimate. Absorption increased but did not reach statistical significance (*p* = 0.055; *g*_rm_ = −0.57). Overall resilience increased (*p* = .04; *g*_rm_ = −0.65) and perceived stress decreased (*p* = 0.04; *g*_rm_ = 0.64) significantly but both the confidence intervals crossed zero, indicating that the effect-size estimates were imprecise and should be interpreted cautiously. Overall, the findings suggest meaningful work engagement changes following the intervention.

**Table 4:** Outcome measures: burnout, stress, work engagement and resilience.

Measures	Pre Mean (SD)	Post Mean (SD)	t (df =7)	Hedges’ *g*_rm_ (r = 0.50)^a^	95% CI for *g*_rm_*	*p*-value
MBI emotional exhaustion	28.13 (11.88)	26.25 (9.02)	1.026	0.322	[-.326,.949]	.169
MBI personal accomplishment	22.38 (3.07)	20.88 (1.96)	1.403	0.441	[-.229, 1.083]	.102
MBI Depersonalisation	11.75 (5.20)	10.25 (4.1)	.929	0.292	[-.351,.916]	.192
Work engagement (UWES overall)	39.5 (10.36)	43.5 (8.5)	−2.530	−0.794	[−1.51,-.037]	.020
UWES Dedication	12.5 (4.38)	14.5 (3.38)	−2.733	−0.858	[−1.596, -.082]	.015
UWES vigor	12.5 (3.55)	13.63 (3.25)	−2.183	−0.685	[−1.377,.041]	.033
UWES absorption	14.5 (2.98)	15.38 (2.26)	−1.825	−0.573	[−1.239,.125]	.055
CDR overall	25.25 (7.7)	30.38 (4.53)	−2.065	−0.649	[−1.33, 0.69]	.039
Perceived stress (PSS)	21.75 (3.45)	20.00 (3.85)	2.033	0.638	[-.076, 1.319]	.041

^*p^ < 0.05 All significance tests were evaluated using one-tailed *p* values based on *a priori* directional hypotheses. MBI, Maslach burnout inventory; PSS, perceived stress scale; UWES, utrecht work engagement scale; CDR, Connor–Davidson resilience scale. *N* = 8. Degrees of freedom for all paired-samples *t* tests were 7.

a Hedges’ *g*_rm_ represents the repeated-measures effect size calculated assuming *r* = .50. Positive and negative values reflect the direction of change according to the scoring of each measure. The 95% confidence intervals for Hedges’ *g*_rm_ are two-sided and are presented to indicate the precision of the effect-size estimates. An interval crossing zero indicates that the effect would not meet a two-sided 5% significance criterion and that its magnitude and direction remain uncertain.

Given the low number of participants, T3 and T4 survey data collections were abandoned. Several factors informed this decision. Firstly, obtaining T2 data already required extensive follow-up, including multiple reminders to participants. Secondly, our partner organisation reported that recent regulatory changes, including the introduction of mandated care minutes per resident, were contributing to significant workforce shortages and placing additional strain on staff. As such, follow up T3 and T4 surveys posed additional ethical and logistical challenges which could not be justified for the small amount of data that would have been collected. Similarly, organisational data on staff sick leave and turnover, which had been intended as indicators of program effectiveness (at T3 and T4), were not collected. With such a small participant group, these measures were unlikely to provide meaningful insights and would have increased the reporting burden on our partner organisation without sufficient benefit to the project. T3 qualitative data were collected, with four MIM participants volunteering to provide feedback through interviews, and to share their reflections on the program. This data is reported below.

### 3.2 Qualitative data

Qualitative findings were drawn from T3 interviews with four MIM participants and post-program meetings with six MAG members. Three overarching findings were identified: perceived personal and professional benefits; generally high acceptability among participants; and implementation barriers that reduced feasibility and appropriateness. Table 5 presents the roles of the MIM participants and MAG members whose perspectives are reported below.

**Table 5:** Interview participant details.

Participant ID	Role
MP1	Enrolled Nurse
MP2	Personal Care Worker
MP3	Enrolled Nurse
MP4	Administration staff member
MAG1	Clinical Nurse Manager
MAG2	Operations Manager
MAG3	Work Health and Safety Advisor
MAG4	Personal Care Worker—Team leader
MAG5	Work Health and Safety Advisor
MAG6	Personal Care Worker—Team leader

#### 3.2.1 Perceived personal and professional benefits of the MIM program

The MIM participants and the MAG members described the MIM program as having a range of personal and professional benefits including greater calm, enjoyment, and improved wellbeing. For example, MP1 reflected: *l just loved the program* *..* *I looked forward to it every morning … and I felt I had got this real deep inner-peace*”. MP4 reflected on her increased gratitude for what she described as the *simple things* in life. MP2 highlighted two key outcomes: mindful eating and the walking meditation. She stated: *I've been doing regular, minimum 30 min of walking meditation … That is the best outcome… [I feel] really relaxed myself after doing that.'* (MP2)

MAG1 notedthat the “*most impactful”* aspect of the program was of creating a daily mindful practice: *I established that (routine). So every day. It's something I do every morning.*

Participants reported improved sleep associated with techniques they learnt in the program:

I certainly got [a lot] out of the breathing techniques, ‘cause I was [a] terrible sleeper …. But I seem to not have any problems with that anymore. (MP3)

“I just now open my bedside drawer, mentally put things in there… Go to bed… and it does seem to work. ‘Cause. Now, I just got to sleep straight away. (MAG4)

I would lie there (in bed) just worrying about things… but then I just started the breathing technique… and I now quite easily do not even think about work when I go to bed anymore. (MAG2)

The program also influenced how participants approached their work. MP4 explained that it encouraged her to “*respond and not react”* when making decisions and take a more mindful approach to her work:

[I don't] just keep on ploughing on doing with what I'm doing…I struggle to slow my brain down… [MIM] brought it to the forefront to say, ‘Okay, get up and go for a walk outside. (MP4)

MP1 also reflected on how the MIM program had encouraged a slower, more mindful pace of work, enabling her to connect more with residents in the service:

what I thought was memorable was the connection component. So what I'm doing is when I stop and ask a resident how they're doing, instead of me walking past them fast asking, I stop and I look them in the eye, and it makes me feel better too, not just them…. I make sure it's a daily practice.

Several MAG members described adopting similar mindful strategies to manage workplace stress after having completed the MIM. MAG1 explained, *[I]don't try and suppress it (stress), just let it come, just let it go”.* Similarly, MAG6 reported the MIM program had assisted them to think about stressful things in a *different way… it does sort of open your mind [I feel] more calm and not stressed.*

#### 3.2.2 Acceptability of MIM program

Participants were asked if they considered whether the MIM program was an acceptable innovation for addressing workplace burnout and associated stress. All participants answered in the affirmative with comments such as: *…absolutely (acceptable)* (MP2)*; what was covered in the sessions was really good* (MP3)*; I loved the whole program. I just loved it… It's a shame more staff haven't taken it on board* (MP1)*.*

Like the MIM participants, the MAG members also reflected on the suitability of the program for the workforce needs. For example: MAG5 stated: “*To be fair, I thought it [the MIM program] did suit the healthcare setting, I thought it was really applicable to the healthcare setting.”* MAG1 also shared similar positive sentiments “*I think it's a great program personally, I’m sold on the program”*.

However not all MAG members found the program acceptable. MAG3 acknowledged that the program did not resonate with her despite the potential benefits: *it would potentially be of benefit if I did use, but I just don't… during the sessions themselves I found them to be interesting… but it's not something that I was massively gravitated to.”*

#### 3.2.3 Mixed method synthesis

Overall, MIM was perceived as acceptable and relevant by most participants and MAG members, although it did not resonate equally with all staff. The low uptake may therefore partly reflect differences in staff perceptions of the program's appeal. Among those who engaged, accounts of greater calm, better sleep, regular mindfulness practice and more deliberate interactions at work were consistent with the directional improvements in quantitative measures of perceived stress, resilience and work engagement. This convergence suggests potential individual benefit among those who participated. This convergence suggests potential individual benefit among those who engaged.

#### 3.2.4 Barriers to feasibility and appropriateness

Despite these positive views about the acceptability of the MIM program, participants identified a range of factors that limited staff engagement with the MIM program. Staffing shortages, workloads, perceptions about feasibility of backfill, limited middle-management engagement, communication barriers, and competing priorities all collectively undermined the program's feasibility and appropriateness.

##### 3.2.4.1 Workload and staffing pressures

MIM participants identified heavy workloads and staff shortages as the primary reasons for low staff participation in the program. MP3 referred to *busy staff schedules* as a key barrier. MP2 elaborated: *Time is precious. We rarely get time to sit down even to have a rest at work, it's so busy.* Similarly, MP1 explained that staffing shortages exacerbated the pressure on already stretched staff: “*There's just no way staff could’ve taken an hour off to do it [MIM program]. … most staff do doubles [double shifts] every day now. It's just horrendous, the staffing levels.”* Participants also noted that, given competing priorities, staff were unlikely to engage unless attendance was mandatory. MP1 explained: “*If it's not mandatory people would not take it seriously, no.”*

Likewise, MAG members echoed similar concerns about feasibility, noting that although the program was well received by staff the combination of workloads and staff shortages impacted staff willingness to participate. As MAG3 explained: “*Carers thought it (MIM program) would make their day harder to attend. As much as they wanted to [attend the MIM program], fitting it into the day would make it [their day] harder”*. MAG 2 shared similar sentiments: “*Staff feel like they don't have time to go to it [MIM program].”* Reflecting on the feasibility of the program, MAG1 explained that for those staff who did enrol in the program, fluctuating workplace pressures contributed to participant attrition:

“From one day to the next, you just don't know what's going to be happening. You could be flat out one day and then probably not as flat out the next… We started off with 10 [staff attending one of the MIM programs], and then they [staff] start dropping like flies.”

##### 3.2.4.2 Staff perceptions about backfill

Staff perceptions about the likelihood of backfill for program attendance, considering current workforce shortages, also emerged as a barrier. MP1 recalled: *‘I would talk [to staff] about it [the MIM program]… and they all would say, “I would've liked to have done that”’.* MP1 went on to explain how this interest was tempered by concerns about the feasibility of their shifts being covered: ‘*Based on past experience they (staff) didn't expect to be supported (backfilled)’.* Similarly, MP2, a personal care worker, highlighted that despite assurances of backfill, staff remained concerned that attending the MIM program would ultimately create more work for their colleagues:

On the floor we are mostly short-staffed and if one person is busy with the… [MIM] program for an hour, then there will be a load for the other care staff on the floor.

Moreover, MP2 suggested that staff would struggle to apply learnings from the MIM program within their already demanding workloads. This data speaks to concerns about the appropriateness of the intervention, and the perceived inability to “fit” another activity into existing workflows:

We have a workload which we have to finish by the time. … I think it'll not come into their [staff] mind to do those [MIM] practices in the workplace. (MP2)

##### 3.2.4.3 Communication and middle management advocacy

Beyond concerns about the feasibility of participation, staff engagement was also shaped by how the program was communicated and promoted. Although email was identified during MAG co-design meetings as the main organisational communication method, MIM participants questioned its effectiveness. MP3 observed that “a lot of them [staff] don't even read them [emails],” while MP2 explained that staff might ignore messages they did not perceive as important: “If that's not important, they won't read the whole letter.”

Face-to-face awareness sessions were included in the implementation plan to address communication silos. However, MAG members reported limited attendance and little encouragement from middle managers for staff to attend. Although the CEO had briefed middle managers about the project, support was not consistently translated into advocacy at the service level. MAG1 explained, “From a top-level leadership perspective, it was only really [the CEO] that was selling it,” and later emphasised, “We couldn't even get people turning up for the information sessions.”

MAG5 similarly suggested that greater middle-management engagement may have enabled staff to attend MIM sessions:

If the managers were more engaged, they would have maybe worked with those team members to help them get to those other training [MIM] sessions.

These findings suggest that the communication problem was not simply an overreliance on email. Program information also lacked consistent reinforcement from managers who could signal its importance and facilitate participation. As MAG5 concluded:

Unless [staff] have someone who's in a direct report-to position [a manager] who can help and support them to get there [to the MIM program], and continue to get there, they're going to drop off.

##### 3.2.4.4 Timing of MIM session and competing priorities

The timing of the MIM sessions created practical challenges in terms of the “fit” of the program with workflows. When co-designing the implementation plan, MAG members specifically chose session times around shift change over time (7 am and 2 pm) to make sessions accessible to as many staff as possible. MAG5 explained “*we tried to pick a variety of times that would suit everyone*.” However, the 2 pm session was identified as problematic. MP2 stated: “*I used to work from 7:00 to 2:00 and the program started at 2:00. I usually get [there] late to join*.” MP1 explained that for frontline staff (carers and nurses) who finish their shift at 3 pm, attending the 2pm session put staff “*on edge the whole time they’re doing the session (because) they would still have to go back and do handover*.” She added: *That's why I didn't respond at first because I thought, ‘Oh no, how's that going to fit in?* (MP1)

Participants also described competing priorities outside work. MP1, MP4 and MP2 all identified that personal care workers often worked at more than one service for financial reasons. MP2 explained, “*most (personal care workers) … not only work in the one place… multiple workplaces. Their priority is… wealth.”* Reflecting on these circumstances MP4 framed staff interest in the program as a question of priority amongst other demands:

…looking at our workforce, we’ve got a lot of workers on the floor, carers and nurses that have busy schedules. They have multiple jobs as well … Have you really got the time to then sit [in the MIM program]?

### 3.3 Mixed-methods synthesis

The qualitative findings provide context for the quantitative pattern of low adoption and inconsistent attendance. Both MIM participants and MAG members identified staffing pressures, doubts that backfill would be available, poor fit of session timing and length with existing workflows, limited middle-management advocacy and communication barriers as factors constraining engagement. These accounts help explain the reduction from 22 registrations to 11 staff commencing the program and the variable attendance across the eight sessions. Taken together, the findings show that, although MIM was perceived as acceptable and beneficial by those who participated, its feasibility and appropriateness were constrained by workforce and organisational conditions.

## 4 Discussion

### 4.1 Key findings

To our knowledge, this Type 2 hybrid study represents the first attempt to implement the MIM program within an aged care organisation. The study was underpinned by the two hypotheses. that participation in the MIM program would: 1) reduce perceived stress and burnout and increase resilience and work engagement, and 2) reduce sick leave and turnover. Low adoption of the MIM program, including low registration, participant attrition and highly variable attendance, meant that Hypothesis 1 could only be partially evaluated and Hypothesis 2 could not be evaluated as specified. The exploratory pre-post analysis (*n* = 8) and qualitative accounts nevertheless indicated possible benefits for staff who engaged, including improved work engagement, resilience, calm, sleep and mindful work practices. However, a larger, adequately powered sample will be required to determine whether the benefits reported in other healthcare settings (22, 27) can be replicated in the aged care context. The principal contribution of our study findings is to show that, despite intervention acceptability among participants, stakeholder co-design and theoretically informed implementation strategies, contextual barriers can suppress adoption and constrain implementation activities.

### 4.2 Interpretation and comparison with the literature

Distinguishing intervention failure from implementation failure is important when an intervention does not produce the expected outcomes (45). Within the aged care literature, studies examining implementation breakdown or incomplete implementation have similarly identified low reach, poor adoption and strong contextual barriers. For example, in an Australian trial, the EDDIE + program was implemented in 11 residential aged care homes to help staff identify and manage early resident deterioration and reduce unnecessary hospital transfers (46). Although the intervention was highly acceptable, implementation fidelity varied, and the expected improvements in hospitalisation-related outcomes were not observed. Workforce shortages, high workloads and a vacancy in the local clinical facilitator role disrupted delivery, despite substantial pre-implementation planning (46). This closely parallels our findings that an intervention can be acceptable and perceived as beneficial yet remain difficult to implement when workforce pressures prevent implementation strategies from being enacted as intended.

Comparable findings were also reported in England in DCM EPIC trial, which evaluated Dementia Care Mapping, a staff-led practice-development process intended to improve person-centred dementia care (47). Seven of the 31 intervention homes did not complete a full intervention cycle, and only four completed all three cycles to an acceptable level. Similar to our findings, staffing shortages limited organisational capacity to provide protected time, intervention complexity and variable management support were found to constrain implementation (47). Similarly, in the Netherlands, the Healthy Working Approach was implemented to improve the sustainable employability of long-term-care workers by supporting teams to identify workplace problems and develop tailored action plans (48). The intervention reached only 11% of teams, although fidelity, satisfaction and perceived benefits were generally positive among the teams that participated (48). This mirrors the combination of very low reach and positive participant experiences in our study and reinforces that acceptability and perceived benefit among those who participate do not necessarily establish organisational feasibility.

Against this literature, our CFIR-mapped findings add three key insights. First, very low recruitment may signal insufficient organisational readiness for implementation. Limited leadership engagement was identified during co-design as a potential barrier within the CFIR construct of Readiness for Implementation, yet strategies to engage middle managers were insufficient to support recruitment. Leadership support is recognised as essential to implementation in aged care (29–31). Although CEO support was identified in the codesign meetings as a major enabler, top-level endorsement did not substitute for active site-level managers who could promote the program and support participation. Organisational readiness is therefore not only a matter of formal leadership endorsement. Insufficient site-level leadership engagement may also reflect organisational culture, which shapes what is prioritised, how decisions are made and how staff and managers respond to change (49). Even well-designed strategies may fail when they do not align with everyday organisational norms and routines (49). In our study, CEO support signalled commitment, but did not translate consistently into a site-level culture in which managers legitimised, prioritised and supported participation in MIM. More targeted middle-management engagement and clear accountability were therefore needed. This is consistent with broader evidence that middle managers translate strategy into practice, address frontline barriers and shape implementation climate (50). Our findings show how insufficient middle-management engagement, operating within organisational culture, can undermine readiness for implementation in residential aged care.

Second, Outer Setting pressures can be expressed through Inner Setting constraints that render otherwise plausible strategies, such as paid release and backfill, unreliable. Workload and staffing pressures emerged as pervasive barriers to implementation, as has been reported elsewhere (46, 47). These barriers were mapped to the CFIR inner setting, namely the constructs of available resources, compatibility and relative priority. However, despite co-designed strategies aligned with the ERIC framework (51), such as rostering staff to attend the MIM program for one hour per week during work hours and funding backfill, these strategies did little to mitigate the barriers. Sector-wide workforce shortages (6), ongoing regulatory reforms and increased regulatory requirements (52–54) have placed Australian aged care organisations under considerable staffing and financial pressure (55, 56). These pressures are consistent with the CFIR Outer Setting construct of External Policy and Incentives. Although these pressures were beyond the remit of our implementation project, they exerted persistent effects on the Inner Setting and limited the feasibility of implementation and the fit of the MIM program in routine practice. Thus, while reimbursing staff and resourcing their time to attend education have been reported as key strategies to support innovation in aged care (29), our findings suggest that these strategies may be insufficient when readiness for implementation is constrained by an operating environment in significant flux. These findings demonstrate a key paradox: the very conditions (chronic staff shortages, heavy workloads, and workplace stress) that create the need for wellbeing interventions such as the MIM program, are also conditions that limit feasibility of participation.

Third, co-design can identify important implementation determinants and guide strategy selection, but it cannot fully anticipate how strategies will be interpreted or enacted in practice. For example, the need for backfill was identified and mapped to the CFIR constructs of Compatibility and Available Resources. However, staff scepticism that backfill would actually be available emerged as a barrier only after implementation commenced. A similar pattern emerged when scheduling the MIM sessions. Holding sessions at shift changeovers was intended to embed the program within existing workflows and maximise staff access, thereby strengthening Compatibility. However, beliefs about unfinished work and a sense of obligation to colleagues took precedence and reduced the fit of this strategy with routine practice. Likewise, reliance on email to raise awareness of MIM reflected a taken-for-granted assumption that disseminating information through established organisational channels would support program visibility and promote participation. Taken together, these factors reduced feasibility and likely contributed to MIM being accorded low relative priority by staff, an important determinant of implementation success in aged care (57). These findings demonstrate why implementation in complex systems is seldom successfully achieved through a single, linear effort. Rather, implementation fit should be treated as provisional and reassessed throughout delivery through iterative cycles of implementation, learning and strategy refinement as conditions change across the Inner and Outer Settings. It is also possible that our findings have revealed how tacit norms, taken for granted understandings and beliefs can shape staff receptivity to different ways of working and how effective implementation strategies are. Nilsen and Kirk (49) highlight that organisational norms and assumptions can be difficult for insiders to recognise without deliberate reflection or an external perspective. In retrospect, a more critical and reflexive agenda adopted by the research team may have assisted MAG members to reveal barriers not readily identifiable within the existing organisational cultural conditions.

### 4.3 Implications for practice

Residential aged care should be recognised as a complex implementation setting in which workforce capacity, organisational readiness, leadership, communication, staff beliefs and existing workflows interact. Contextual barriers may be particularly resistant to local change when they are driven by Outer Setting pressures, such as sector-wide workforce shortages, regulatory demands and financial constraints. In these circumstances, strategies such as paid release and backfill may appear appropriate but remain difficult to enact. Before implementation, organisations should undertake a structured assessment of readiness. The aged-care-specific Readiness Assessment Tool developed by Davy et al. (58) offers a practical approach to assessing individual attitudes and capabilities, organisational context, innovation fit, interpersonal connections and proposed implementation strategies. Additionally, readiness and implementation fit should continue to be reviewed during rollout rather than assumed to remain suitable because strategies were co-designed in advance. Low registration, early attrition and inconsistent attendance should be treated as early implementation signals that trigger review and adaptation. More flexible delivery approaches, such as shorter sessions, multiple session times or blended synchronous and asynchronous formats, may improve fit with aged care workflows, although the core components of MIM and fidelity to these components should be specified and monitored. Where minimum conditions such as sufficient staffing and credible protected time cannot be met, adapting or delaying implementation may be more appropriate than proceeding with a plan that staff do not believe can be enacted.

### 4.4 Implications for research

Future research should examine how co-design processes can better identify taken-for-granted assumptions about routine aspects of practice. Although staff bring intimate knowledge of their setting, tacit organisational norms and routines may be difficult for insiders to recognise (49). Research should therefore explore possibilities for more critical interrogation of implicit beliefs that may contribute to unidentified barriers. This cultural analysis may then open the way for more reflexive and transformative co-design engagement (59) and reveal previously invisible barriers to change. Future studies should also use more adaptive implementation strategy designs (60) that allow strategies to be reviewed and modified as emerging barriers encounter frontline workflows. Predefined thresholds for low recruitment, attendance and retention could be used to trigger real-time review and adaptation rather than waiting until implementation has concluded to determine that strategies were not working as anticipated. Finally, larger, multi-site studies are needed to determine whether the benefits of MIM reported in other healthcare settings can be replicated in residential aged care. Future studies should compare the standard eight-week synchronous format with shorter, blended or asynchronous delivery models while preserving and monitoring the program's core components.

### 4.5 Limitations

This study has several limitations that should be considered when interpreting the findings. First, the small sample of staff who provided pre- and post-program survey data limited statistical power, and the effectiveness estimates should be interpreted with caution. Second, most qualitative data were drawn from a small group of engaged participants and MAG members and may not represent the broader workforce. We did not interview staff who declined to enrol; perspectives on fit, communication, feasibility and competing priorities are therefore incomplete. Third, implementation occurred in a single aged care organisation, and features such as leadership structure and organisational culture may limit transferability. Fourth, combining paired data from MAG members and Stage 3 participants increased the number of observations but brought together staff who completed MIM at different stages and under different participation conditions; the estimates should therefore be regarded as exploratory. Fifth, voluntary participation may have preferentially attracted staff already interested in mindfulness or convinced of its benefits, creating self-selection bias in both the quantitative and qualitative findings.

## 5 Conclusion

Implementation breakdown, rather than evidence of intervention ineffectiveness, was the central finding of this study. MIM was acceptable and perceived as beneficial by staff who participated, but low adoption prevented a robust test of the project hypotheses. In complex care settings, acceptability, co-design and theoretically matched implementation strategies may be insufficient when organisational readiness is constrained by limited staffing capacity, unreliable protected time, insufficient middle-management engagement and ineffective communication. Future implementation efforts should assess readiness before rollout and continue to monitor and adapt strategies as contextual conditions change. Further trials should test more flexible MIM delivery models while preserving core components and measuring both effectiveness and implementation outcomes.

## Data Availability

The raw data supporting the conclusions of the study will be made available upon reasonable request to the corresponding author and in accordance with the study's ethics approval.
